# Specific plasma amino acid disturbances associated with metabolic syndrome

**DOI:** 10.1007/s12020-017-1460-9

**Published:** 2017-10-26

**Authors:** Marta Siomkajło, Jacek Rybka, Magdalena Mierzchała-Pasierb, Andrzej Gamian, Joanna Stankiewicz-Olczyk, Marek Bolanowski, Jacek Daroszewski

**Affiliations:** 1 Department of Endocrinology, Diabetes and Isotope Therapy, Wroclaw Medical University, L. Pasteur 4, Wroclaw, 50-367 Poland; 2 Laboratory of Medical Microbiology, Hirszfeld Institute of Immunology and Experimental Therapy, Polish Academy of Sciences, R. Weigl 12, Wroclaw, 53-114 Poland; 3 Department of Medical Biochemistry, Wroclaw Medical University, Chalubinskiego 10, Wroclaw, 50-368 Poland

**Keywords:** Aminoacids, Metabolic syndrome, BCAA, Phenylalanine, Diagnosis

## Abstract

**Purpose:**

The primary objective of the present study was to examine the association between branched chain and aromatic amino acid profiles (BCAA and AAA respectively) and the metabolic syndrome (MS), and to evaluate the clinical utility of these associations in the diagnostic process.

**Methods:**

Two hundred and sixty three healthy men with MS [MS(+): *n* = 165] and without MS [MS(−): *n* = 98] were enrolled in the observational study. Anthropometrical, biochemical, and amino acid measurements were performed. The ability of the BCAA and AAA to discriminate subjects with MS and insulin resistance was tested. Based on logistic discrimination, a multivariate early MS diagnostic model was built, and its discrimination properties were evaluated.

**Results:**

Two functionally independent amino acid clusters were identified. BCAA and phenylalanine differed significantly between MS(+) and MS(−) participants (*P* = 0.003). These factors were also found to be indicators of MS(+) individuals (AUC: 0.66; 95% CI: 0.5757–0.7469), and correlated with cardiometabolic factors. No statistically significant differences in amino acid concentrations between those with and without insulin resistance were noted, and none of the amino groups were indicators of insulin resistance. The proposed MS multivariate diagnostic model consisted of phenylalanine, insulin, leptin, and adiponectin, and had good discrimination properties [AUC 0.79; 95% CI: 0.7239–0.8646].

**Conclusions:**

MS is associated with selective BCAA and AAA profile disturbances, which could be part of cardiometabolic disease pathogenesis and derive neither directly from insulin sensitivity impairment, nor obesity or muscle mass. The MS diagnostic model developed and described herein should be validated in future studies.

## Introduction

There is a great need for novel MS biomarker that would allow for early identification of those at greatest cardiometabolic diseases (CMD) risk as well as individual metabolic risk stratification and monitoring, so that appropriate intervention strategies could be developed and implemented [1–3]. Dichotomization of the currently used MS criteria limits their application in daily practice.

Current evidence coming from studies across numerous ethnic backgrounds supports use of branched chain amino acid (BCAA) and aromatic amino acid (AAA) profile as biomarkers determining metabolic health. The BCAA include leucine (Leu), isoleucine (Ile), and valine (Val), and the AAA, phenylalanine (Phe), tryptophan (Trp), and tyrosine (Tyr) [4–7].

Close association between changes in BCAA and AAA profiles with CMD: diabetes type 2 (DM2), cardiovascular disease (CVD), insulin resistance (IR) and obesity have been demonstrated [4–9]; specifically their diagnostic and prognostic value, correlation with positive outcomes of therapeutic interventions, and ability to differentiate metabolically healthy from metabolically unhealthy obese patients [4, 9]. There is also accumulating data demonstrating that this association is independent of traditional risk factors, enhances the prediction value, and could be useful for relative CVD risk assessment [10, 11].

BCAA, Phe, and Tyr have been proved to be predictive of DM2 [12, 13] and CVD [11] up to 12 years prior to disease manifestation, BCAA and Tyr were related with MS diagnosis within 4-year period [13], whereas BCAA and Phe were shown to be significantly positively correlated with IR values measured 18 months later [14]. These observations suggest that some disturbances of BCAA and AAA metabolism could be part of an early, yet to be elucidated, metabolic change that precedes CMD development, and links DM2 and CVD pathogenesis [11]. That makes BCAA and AAA an auspicious candidate for new, early MS biomarkers.

Metabolomic analysis of nearly 1900 individuals from three different American cohorts identified BCAA, Phe, Tyr, and three other amino acids (AA): Met, Ala, His as the variables that out of 55 assessed metabolites differed most between metabolically healthy and unhealthy study participants, and their significant discriminant power, independent of BMI, was documented [5]. In Finnish study, BCAAs, Phe, and Tyr, in combination with the inflammation protein orosomucoid, allowed for the discrimination of the overweight/obese women with the MS from those that were metabolic healthy, irrespective of age, body mass, fat mass, physical activity. The distinguished metabolites were also correlated with MS factors [15].

The concept of BCAA and AAA profile alterations being indicative of metabolic disorders has a deep pathophysiological origin. Changes in the availability of BCAA and AAA have a profound effect on cell signaling, gene expression, brain, and neuroendocrine function [16, 17]. In addition, BCAA availability affects glucose, protein, and lipid metabolism [18]. Some AA-transporter systems are considered to have a dual role, as both a transporter and a receptor. In this role, the ‘transceptor’ communicates information about nutritional state, as well as quantity and quality of extra and intracellular AAs, to nutrient-sensitive factors such as GCN and mTOR, which enables the cell to respond appropriately to change [17]. These factors can be also involved in pathophysiological effects; in that, under certain conditions, BCAA are considered to be IR-promoting factors by way of mTOR, AMPK, and GCN2 overstimulation [19, 20].

The competitive nature of some of the BCAA and AAA transmembrane transporters determines the functional interdependence of individual AA concentrations; thus, AA proportions, rather than the absolute AA concentrations alone could be of clinical significance [21, 22]. Plasma molar ratios of individual BCAA and AAA to the rest of the competitors predict individual AA uptake through the brain blood barrier [23].

The central aims of the present study were to: (1) examine the association between the profiles of BCAA and AAA and the phenotype of MS, as well as the pathogenic factors of MS (IR and obesity); (2) evaluate the diagnostic value of BCAA-concentrations and AAA-concentrations; and, (3) assess the validity of using BCAA and AAA measurements for diagnostic modeling of early MS.

## Methods

### Study participants

Two hundred and sixty-three Caucasian men, aged 36–60 years, with neither a history of diabetes, nor of lipostatic, diabetic or psychiatric drug therapy. The participants were recruited from 290 randomly selected employees of a Polish company, who performed manual or desk-based work. The participants were allocated to one of two groups, either MS(+) (*n* = 165) or MS(−) (*n* = 98), based on their fulfillment of the modified definition of MS (Joint Interim Society statement [24]), with waist circumference (WC) replaced with waist-to-height ratio (WtHr) [19, 20]. At least three of the following criteria needed to be met for inclusion in the MS(+) group: WtHr > 0.5; triglyceride (TG) level ≥ 1.7 mmol/L; high-density lipoprotein (HDL) level < 1.03 mmol/L; systolic pressure (SYS) ≥ 130 mmHg/diastolic (DIAS) ≥ 85 mmHg/hypotensive treatment; and, fasting glucose (FG) level ≥ 5.6 mmol/L.

For further analyses, participants were categorized into groups, based on whether they were insulin resistant [IR(+)], or not [IR(−)], according to homeostatic model assessment of insulin resistance (HOMA IR) values, which were measured using the formula: insulin (µU/mL) × glucose (mmol/L)/22.5. Cut-off HOMA IR values were the same as those determined by Szurkowska et al. [25] for a Polish population. Of the 263 enrolled participants, 124 were classified as IR(+) (HOMA IR > 2.1) and 134 as IR(−) (HOMA IR ≤ 2.1).

### Measurements

Anthropometric and laboratory measurements were performed during periodic medical examinations conducted in 2015.

Weight, height, and waist and hip circumference were determined using standard techniques. Body mass index (BMI), waist-to-hip ratio (WHR) and WtHr were determined as: weight (kg)/height (m^2^); waist (cm)/hip (cm); and, waist (cm)/height (cm), respectively. Bioelectrical impedance analysis (Tanita T6360, Japan) was used to measure body fat mass, fat-free mass and percentage body fat. Lean body mass was calculated as: total body mass – percentage fat mass × body mass. Blood pressure (BP) was measured using a mercury/Aneroid sphygmomanometer, with final BP calculated as the mean of two readings taken from the participant’s non-dominant arm, while seated, after a 20-min rest. Intima media thickness (IMT) was measured using B-mode ultrasound (Philips iU22 ultrasound imager) with a linear array on the posterior wall of the carotid arteries. The final IMT value was the mean of several IMT measurements from the right and left common, internal and external carotid arteries.

Laboratory measurements were performed after overnight fasting, using commercially available methods. Total cholesterol (TC), HDL, and TG levels were calculated using enzymatic measures on a COBAS Integra 400 Plus Analyzer (Roche Diagnostics). Low-density lipoprotein C (LDL-C) was calculated using Friedewald’s formula: LDL = TC – HDL – TG/5 (mg/dL). C-reactive protein (CRP) was measured using an immunoephelometric assay (Dade Behring GmbH, Germany); insulin using an electrochemiluminescent method (Elecsys, Roche); leptin (LEP) using an RIA assay (EMD Millipore Human Leptin, USA) with the range set at 3.8 ± 1.8 ng/mL; and, adiponectin using a RIA assay (EMD Millipore Human Adiponectin, USA) with the limit of sensitivity set at 1 ng/mL.

Plasma samples were analyzed for AAs using gas–liquid chromatography combined with tandem mass spectrometry (GLC-MSMS Focus GC – IonTrap ITQ700 (Thermo) system). The commercially available EZ:faast amino acids analysis kit (Phenomenex, USA) assay was used to analyze AAs in the biological sample. The calibration standards and samples were prepared according to the Phenomenex EZ:Faast(™) kit sample preparation procedure [26]. AAs, after isolation were converted into their volatile derivatives using propyl chloroformate, separated with gas chromatography, and analyzed by tandem mass spectrometry using GLC-MSMS system Focus GC – IonTrap ITQ700 (Thermo).

### AA ratios

For the purposes of the study, several AA molar ratios were analyzed:

Leucine ratio (Leu_IVPTT): molar ratio of Leu to the sum of AAAs, Ile, Val;

- Shortened leucine ratio (Leu_IV): molar ratio of Leu to the sum of Ile, Val.
- Tyrosine ratio (TyrPhe_LIVTrp): molar ratio of the sum of Tyr and Phe to the sum of the BCAAs and Trp.
- Tryptophan ratio (Trp_LIVTyr): molar ratio of Trp to the sum of BCAAs, Tyr and Phe.

### Ethical statement

The study was approved by the local ethics committee (Wroclaw Medical University KB-182/2015), and all participants provided informed consent.

### Statistical analysis

Before statistical analyses were undertaken, a Box-Cox transformation was conducted for variance stabilization. Statistical significance for comparative analysis was set at *P < *0.05. On AA variables, principle component (PC) analysis was performed. PC number was limited by 80% of explained variation. For all AA variables and PCs, correlation analysis with anatomical and biochemical variables, as outlined in the methods section, was performed. Estimations of 95% confidence intervals (CIs) were based on bootstrapping. The median PCs of the MS(+) and MS(−) groups were compared. Statistical significance was evaluated using a permutation test. Logistic discriminant analysis was used to determine the validity of using PC to differentiate MS(+) and MS(−) participants. ROC curve and AUC values were derived. De Long’s method was used to determine the 95% CI.

Logistic discriminant analysis utilizing AAs and the biochemical variables that differed most between the groups was used to create a multivariate discrimination model for MS diagnosis. The ROC curve was derived and the AUC assessed. DeLong’s method was used to determine the 95% CI. Differences testing and discriminant analysis on the PCs were performed for IR(+) and IR(−) groups.

The free R Stats Package version 3.5.0 was used for statistical analyses and figures preparation.

## Results

A comparative analysis of the clinical characteristics and AA concentrations in the MS(+) and MS(−) groups is presented in Table 1. Clear differences between the MS(+) and MS(−) participants, in terms of AA profiles, were observed. The concentrations of individual AAs (Ile, Phe, Leu, and Val), as well as summary concentrations of BCAAs, AAAs, and BCAAs together with AAAs (BCAA_AAAs) were significantly higher in the MS(+) group, when compared with the MS(−) group. No statistically significant differences in Tyr, Trp, and all tested AAs ratios were observed.

**Table 1:** Baseline and AA characteristics of MS(+) and MS(−) groups. Data are expressed in the original scale as medians and robust SD with *P* values for differences testing

Parameter/group	MS(−), *n* = 98	MS(+), *n* = 165	*P* values
WC [cm]	90 ± 7.97	98 ± 5.93	< 0.001
WHR	0.9 ± 0.04	0.94 ± 0.04	0.475
WtHr	0.5 ± 0.04	0.56 ± 0.04	< 0.001
BMI [kg/m^2^]	25.5 ± 2.59	29 ± 2.97	< 0.001
FM%	20.5 ± 5.19	27 ± 4.45	< 0.001
FFM [kg]	63.2 ± 5.01	64.77 ± 5.86	0.063
FM [kg]	16.7 ± 5.41	23.45 ± 6.26	< 0.001
IMT [mm]	0.545 ± 0.074	0.55 ± 0.074	0.852
LDL [mmol/L]	3 ± 0.85	3.3 ± 0.82	0.008
TG [mmol/L]	1.2 ± 0.52	2 ± 0.89	< 0.001
HDL [mmol/L]	1.56 ± 0.38	1.29 ± 0.29	< 0.001
CRP [mg/L]	0.95 ± 0.95	1.67 ± 1.65	0.744
SYS [mmHg]	120 ± 7.41	140 ± 7.41	< 0.001
DIAS [mmHg]	80 ± 6.49	90 ± 3.71	< 0.001
FG [mmol/L]	5.35 ± 0.35	5.8 ± 0.52	< 0.001
insulin [µIU/mL]	5.95 ± 2.49	9.52 ± 5.75	< 0.001
HOMA IR	1.46 ± 0.58	2.51 ± 1.63	< 0.001
ADI [µg/mL]	7.5 ± 4.11	6.5 ± 3.17	0.001
LEP [ng/mL]	5.85 ± 3.47	7.4 ± 3.98	< 0.001
LEP/ADI	0.73 ± 0.54	1.12 ± 0.89	< 0.001
Amino acid concentrations [nmol/mL]			
Leucine	132.8 ± 39.5	157.7 ± 38.5	0.005
Valine	247.3 ± 74.2	272.7 ± 68.9	0.032
Isoleucine	58.8 ± 18.5	69.9 ± 15.7	0.001
Phenylalanine	74.8 ± 19.0	88.6 ± 23.4	0.002
Tyrosine	25.9 ± 9.93	26.95 ± 10.17	0.261
Tryptophan	56.4 ± 24.4	59.3 ± 30.9	0.102
BCAA	433 ± 125	512 ± 115	0.008
AAA	164.1 ± 36.7	176.1 ± 56.2	0.006
BCAA_AAA	589 ± 120	674 ± 133	0.002
Amino acid ratios			
Leu_IV	0.45 ± 0.08	0.46 ± 0.07	0.926
Leu_IVPTT	0.29 ± 0.05	0.31 ± 0.05	0.436
Trp_LIVTyr	0.11 ± 0.06	0.12 ± 0.06	0.982
TyrPhe_LIVTrp	0.21 ± 0.03	0.21 ± 0.03	0.677

*AAA* aromatic amino acids, *ADI* adiponectin, *BCAA* branched chain amino acids, *BCAA_AAA* summary concentration of BCAA and AAA, *BMI* body mass index, *CRP* C-reactive protein, *DIAS* diastolic blood pressure, *FG* fasting glucose, *FFM* fat free body mass, *FM* fat body mass, *FM%* percentage of body fat, *HDL* high density cholesterol, *HOMA IR* homeostatic model assessment of insulin resistance, *IMT* intima media thickness, *LDL* low density lipoprotein, *LEP* leptin, *LEP/ADI* leptin-to-adiponectin ratio, *Leu_IV* shortened leucine ratio, *Leu_IVPTT* leucine ratio, *SYS* systolic blood pressure, *TG* triglycerides, *Trp_LIVTyr* tryptophan ratio, *TyrPhe_LIVTrp* tyrosine ratio, *WC* waist circumference, *WHR* waist-to-hip ratio, *WtHr* waist-to-height ratio

Two AA factors were identified using principal component analysis (PCA), and subsequently named factor 1 (AA1) and factor 2 (AA2). AA1 was determined by the concentrations of Leu, Ile, Val (i.e. the BCAAs) and Phe, and AA2 by the concentrations of Tyr and Trp. Statistically significant but relatively weak correlations between AA1 and cardiometabolic status indicators (i.e. BMI, WtHr, fat body mass (FM), fat body mass percentage (FM%), WC, SYS, DIAS, insulin, quantitative insulin sensitivity check index (QUICKI), HOMA IR, FG, CRP, and LEP) were observed (Table 2). No statistically significant correlations for AA2 were noted. AA1 was observed to differ significantly between the MS(+) and MS(−) groups (*P* = 0.003), whereas AA2 did not (*P* = 0.722). Logistic discriminant analysis confirmed that AA1, unlike AA2, is an indicator of MS(+) status; however, the discriminatory power was not high (AUC: 0.66; 95% CI: 0.5757–0.7469).

**Table 2:** Statistically significant correlations (*R*-value, 95% CI) of identified in PCA amino acid factor 1 (AA1). AA1 was determined by the concentrations of Leu, Ile, Val (i.e. the BCAAs) and Phe

Parameter	r AA1	95% CI AA1
BMI [kg/m^2^]	0.19	0.05; 0.33
WC [cm]	0.18	0.07; 0.31
FM [kg]	0.16	0.02; 0.29
FM%	0.16	0.02; 0.29
WtHr	0.20	0.09; 0.32
SYS [mmHg]	0.15	0.01; 0.3
DIAS [mmHg]	0.17	0.03; 0.33
FG [mmol/L]	0.14	0.03; 0.26
Insulin [µIU/mL]	0.19	0.04; 0.34
HOMA IR	0.20	0.07; 0.32
QUICKI	(−)0.2	(−)0.34; (−)0.07
LEP [ng/mL]	0.18	0.07; 0.31
CRP [mg/L]	0.15	0.02; 0.28

*BMI* body mass index, *CRP* C-reactive protein, *DIAS* diastolic blood pressure, *FG* fasting glucose, *FM* fat body mass, *FM%* percentage of body fat, *HOMA IR* homeostatic model assessment of insulin resistance, *LEP* leptin, *QUICKI* quantitative insulin sensitivity check index, *SYS* systolic blood pressure, *WC* waist circumference, *WtHr* waist-to-height ratio

Analogical analyses for the IR(+) and IR(−) groups were performed, and no statistically significant differences in terms of AA concentrations and AA ratios were observed. BCAAs showed only a trend towards diversification. Logistic discriminant analysis confirmed that neither AA1 nor AA2 is a meaningful indicator of IR(+) status. The characteristics of the groups according to the cardiometabolic indices and AA concentrations with difference testing are presented in Table 3.

**Table 3:** AAs concentrations and baseline characteristics of the IR+ and IR− groups. Data are expressed in the original scale as median and robust SD with *P* values for differences testing

Parameter	IR(−), *n* = 134	IR(+), *n* = 124	P*
WC [cm]	90.5 ± 8.15	96 ± 6.67	<0.001 (*)
FM%	21 ± 4.82	26 ± 5.93	<0.001 (*)
FM [kg]	16.83 ± 5.25	22.25 ± 6.83	<0.001 (*)
WtHr	0.51 ± 0.04	0.55 ± 0.05	<0.001 (*)
FFM [kg]	62.35 ± 5.01	64.17 ± 5.75	0.252
WHR	0.9 ± 0.04	0.93 ± 0.04	0.128
BMI [kg/m^2^]	25.5 ± 3.52	28 ± 2.97	<0.001 (*)
IM [mm]	0.545 ± 0.087	0.548 ± 0.074	0.87
SYS [mmHg]	125 ± 14.83	130 ± 7.41	0.003 (*)
DIAS [mmHg]	80 ± 7.41	90 ± 7.41	0.067
LEP [ng/mL]	4.6 ± 2.35	7.1 ± 3.78	<0.001 (*)
LEP/ADI	0.59 ± 0.37	1.02 ± 0.77	0.003 (*)
ADI [µg/mL]	8.35 ± 4.34	6.9 ± 3.56	0.209
CRP [mg/L]	0.83 ± 0.7	1.41 ± 1.56	0.096
HDL [mmol/L]	1.67 ± 0.35	1.37 ± 0.33	<0.001 (*)
TG [mmol/L]	1.35 ± 0.58	1.7 ± 0.89	0.437
LDL [mmol/L]	3.1 ± 0.8	3.2 ± 0.87	0.189
FG [mmol/L]	5.2 ± 0.39	5.7 ± 0.67	<0.001 (*)
Insulin [µIU/mL]	2.93 ± 0.78	8.6 ± 5.18	<0.001 (*)
HOMAIR	0.7 ± 0.22	2.13 ± 1.56	<0.001 (*)
Amino acid concentrations [nmol/mL]			
Leucine	139.2 ± 30.6	154 ± 42	0.057
Isoleucine	56 ± 13.6	68.1 ± 18	0.068
Valine	239 ± 56.9	262.5 ± 72.4	0.097
Phenylalanine	78.1 ± 18.6	83.3 ± 22.7	0.21
Tyrosine	28.3 ± 6.82	27.2 ± 10.49	0.807
Tryptophan	64 ± 15.6	59.3 ± 29.3	0.828
BCAA	432.6 ± 87.3	477.8 ± 129.4	0.07 (*)
AAA	167.6 ± 27.5	171 ± 49.4	0.888
BCAA_AAA	597 ± 115	655 ± 135	0.297
Amino acid ratios			
Leu_IV	0.46 ± 0.05	0.46 ± 0.07	0.95
Leu_IVPTT	0.29 ± 0.02	0.3 ± 0.05	0.136
Trp_LIVTyr	0.13 ± 0.05	0.12 ± 0.06	0.834
TyrPhe_LIVTrp	0.22 ± 0.03	0.21 ± 0.03	0.621

*AAA* aromatic amino acids, *ADI* adiponectin, *BCAA* branched chain amino acids, *BCAA_AAA* summary concentration of BCAA and AAA, *BMI* body mass index, *CRP* C-reactive protein, *DIAS* diastolic blood pressure, *FG* fasting glucose, *FFM* fat free body mass, *FM* fat body mass, *FM%* percentage of body fat, *HDL* high density cholesterol, *HOMA IR* homeostatic model assessment of insulin resistance, *IMT* intima media thickness, *LDL* low density lipoprotein, *LEP* leptin, *LEP/ADI* leptin-to-adiponectin ratio, *Leu_IV* shortened leucine ratio, *Leu_IVPTT* leucine ratio, *SYS* systolic blood pressure, *TG* triglycerides, *Trp_LIVTyr* tryptophan ratio, *TyrPhe_LIVTrp* tyrosine ratio, *WC* waist circumference, *WHR* waist-to-hip ratio, *WtHr* waist-to-height ratio

**Fig. 1:**
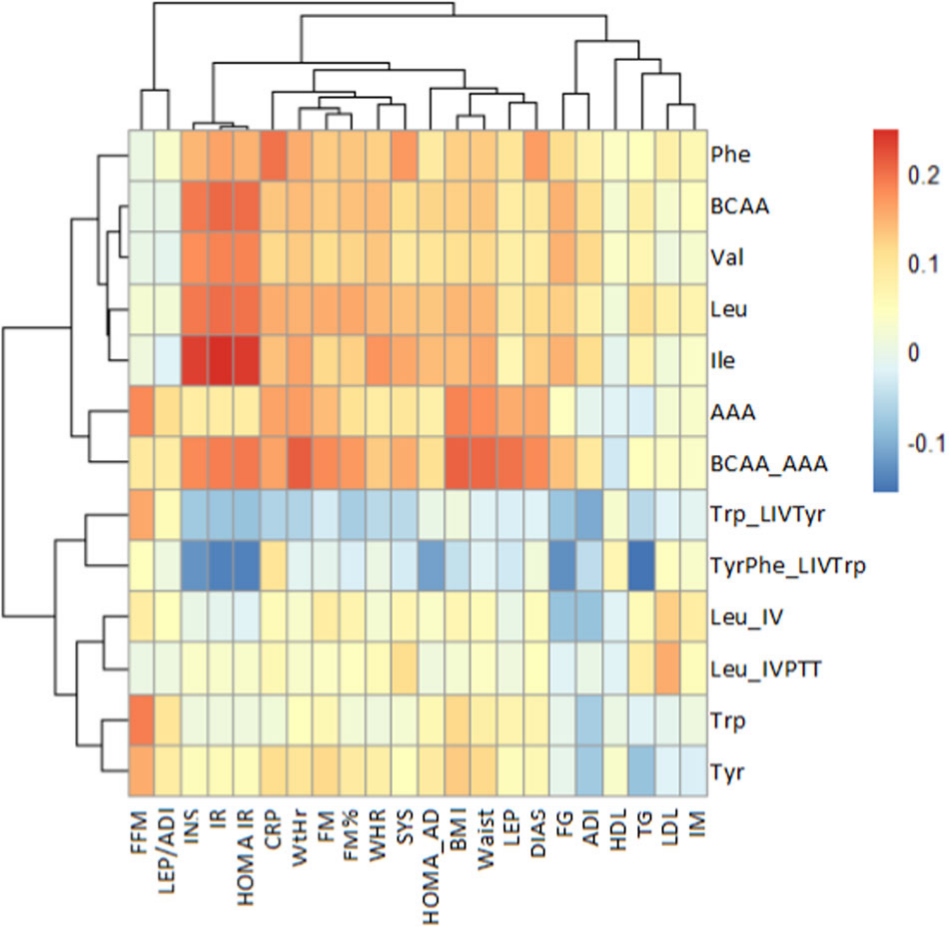
Correlation clustering using amino acid profile, metabolic and anthropometric variables. Clamps present hierarchical clustering of the variables. AAA aromatic amino acids, ADI adiponectin, BCAA branched chain amino acids, BCAA_AAA summary concentration of BCAA and AAA, BMI body mass index, DIAS diastolic blood pressure, FG fasting glucose, FFM fat free body mass, FM fat body mass, FM% percentage of body fat, IM intima media, LEP leptin, LEP/ADI leptin-to-adiponectin ratio, Leu_IV shortened leucine ratio, Leu_IVPTT leucine ratio, SYS systolic blood pressure, TG triglycerides, Trp_LIVTyr tryptophan ratio, TyrPhe_LIVTrp tyrosine ratio, WC waist circumference, WtHr waist-to-height ratio, IR = (-)1 × QUICKI

The correlation structure between assessed AAs and metabolic, anthropometrical and physiological variables is depicted with the aid of hierarchical clustering, in Fig. 1. All observed linear relationships were found to be weak. The association between the IR indices (QUICKI, HOMA IR) and Ile, Leu, and BCAA, as well as between BMI, WtHr, WC, LEP, and BCAA_AAA had more prominent correlation coefficients, but none exceeded 0.25. Notably, neither for Tyr nor Trp were statistically significant linear relationships with cardiometabolic indicators noted. Trp (and its derivatives) was the only AA that showed significant association with fat free body mass (FFM).

By means logistic discriminant analysis, a multivariate model for MS(+) diagnosis, based on Phe, INS, LEP, and ADI was built (*MS screen test*). On the discriminant function value, Phe and INS were strongest (*P* = 0.006 and 0.002, respectively), whereas LEP and ADI were weakest (*P* = 0.1 and 0.06, respectively). The resulting model coefficients for Phe, insulin, and LEP were positive, whereas that of ADI was negative. The AUC value of the classification model was indicative of relatively good classification properties (AUC: 0.79; 95% CI: 0.7239–0.8646).

## Discussion

The principal aim of the present study was to examine the association between BCAA and AAA profiles and MS phenotype, and to search within those AAs for a biomarker of early MS. We demonstrated that BCAA and AAA can be used to differentiate MS(+) and MS(−) subjects. Two distinct and functionally different AA groups were identified: the first comprised BCAA and Phe (AA1), and the second, Tyr and Trp (AA2). AA1 differed significantly between MS(+) and MS(−) participants, had significant discriminant power for MS(+) individuals and was correlated with the typical cardiometabolic disturbances associated with the MS phenotype (albeit in a weak manner). On the contrary, AA2 showed neither a statistically significant difference between the MS(+)/MS(−) groups, nor was it able to discriminate MS(+) individuals. Furthermore, no statistically significant correlations with cardiometabolic disturbances were observed.

The results presented herein, which highlight BCAA and Phe, are consistent with observations about related to cardiometabolic disturbances essential selective hyperaminoacidemia [5, 12, 13].

### AA profile and IR

Considering the known association between IR and AA profile [8, 27], as well as the effect of insulin on intracellular AA transport [28] and activity of main, rate-limiting enzyme of BCAA catabolism (BCKDH) [29], we performed additional analyses to test whether differences in BCAA and AAA concentration were the result of insulin sensitivity impairment. We failed to find an association between assessed AA concentrations and IR in the context of glucose metabolism. The AA concentration did not differ between the groups with higher and lower HOMA IR values, nor did it allow for the discrimination of the IR+ group. All correlation coefficients values were very weak. Our results could be affected by relative low HOMA IR distribution values as it was suspected in the study of Newgard et al. [8], especially considering that HOMA IR distribution values in the groups of our study were even lower than that of Newgad’s.

IR is multidimensional, in that it affects different tissues to different degrees. Therefore, the aforementioned observation does not rule out the possibility that BCAA and AAA profile is associated with other aspects of IR. In fact, MS itself seems to be the manifestation of selective insulin transmission [30–32]. A disturbance in AA profile could also be an early IR manifestation that is seen only in the metabolome and precedes changes in traditional insulin sensitivity indicators [12, 13, 33, 34].

### AA profile and obesity

An additional objective of the present study was to elucidate the association between BCAA and AAA profile and obesity. BCAA are metabolized mainly in the muscle and adipose tissue [35, 36]. Newgard et al. [8] demonstrated increased concentration of BCAA and metabolites in obese compared with lean individuals and this measure had significant discriminant power. The authors stated the relationship likely derived from overnutrition and increased metabolite flow through the catabolic pathways. In other studies, decreased expression and/or activity of BCAA-degrading enzymes in the adipose tissue was observed in obese, T2DM and IR individuals [37], and was associated with metabolic disturbances that improved after weight loss [36–38]. Metabolically unhealthy individuals, as compared to metabolically healthy subjects, had significantly lower expression of genes associated with BCAA catabolism [38].

The results of the present study failed to find evidence in support of a close association between BCAA and AAA profile disturbances and obesity. In previous work BCAA were proposed as indicators of ‘normal-weight obese individuals’ and demonstrated to be markers of VF but not SF [39]. The lack of an objective measure of VF in the present study might be the reason that only relatively weak correlations were observed.

Our results also suggest that an increased concentration of BCAA is not directly correlated with increased body mass, nor with muscle mass (the body’s primary source of AAs). Trp was the only AA correlated with FFM, but not with cardiometabolic disturbances; this result could be driven by an albumin-related fraction unique to Trp that determines the different effects of insulin [40].

### Selectivity of hyperaminoacidemia

As demonstrated in the present and other studies, hyperaminoacidemia is selective. If the catabolic pathways of BCAAs and AAAs are separate, why then are BCAA observed to cluster with Phe (as in the current study) or with both Phe and Tyr (in most other studies) [5, 11–15]. It should be noted that Tyr and Phe are closely linked since Tyr is synthesized from Phe [40]. Of the AA-degrading enzymes, only the BCKDH complex and phenylalanine hydroxylase are regulated by phosphorylation and dephosphorylation [29, 40]. As such, they are key targets for the action of insulin.

Another explanation for the clustering could lie in the competitive character of the transmembrane transport system common to both BCAA and AAA [21]. The elevation of AAA has been suggested to be secondary to the increase in BCAA concentration [8]. The pattern of AA profile alteration in CMDs reflects the selectivity and preference of the main AA transporters [29, 41].

### BCAA and AAA ratios

The BCAA and AAA ratios, as the predictors of individual AA uptake have been widely studied [22, 23, 42, 43] and linked to neurobiological origin of obesity and metabolic diseases [44, 45]. That encouraged the analysis of AA ratios in MS+and MS− groups. Trp affects serotonergic neurotransmission that plays a role in regulation of food intake, body weight and mood [22–24, 42, 43]. The dopaminergic pathway relies on Tyr and Phe supply and is involved in food-based reward as well as is most closely linked to hunger [46]. Leu has been linked to nutrient sensitive hypothalamic neurons that affect behavioral and physiological determinants of energy balance [45]. Leu function are unique also in that it promotes insulin secretion, has a profound effect on mTOR stimulation and lowers the concentration of other BCAA (Val, Ile) [47].

The present study failed to find evidence in support of a relationship between AA ratios and MS. It is likely that AA ratios become important only in the postprandial stage, when the plasma concentration of AAs is increasing, and the competition for transporters is higher. In contrast, Newgard et al. [8] reported a significantly lower Trp ratio and unchanged median Trp levels in obese, as compared to lean, individuals.

### Utility of AAs in the diagnosis of MS

Whether BCAA are passive markers of metabolic disturbances or—consistent with much of the research published to date—they facilitate them, is yet to be fully elucidated [4]. The differences between the MS(+) and MS(−) groups in terms of BCAA and AAA concentration, which neither resulted from impaired glucose metabolism nor meaningful correlated with cardiometabolic factors, suggest that a disturbance in AA profile may be the result of other processes involved in CMD pathogenesis. Our study group consisted of apparently healthy subjects who did not display overt symptoms of CVD, so we can assume that the abnormalities observed in these patients are indicative of the early-stage pathophysiological events.

In our study, BCAA and AAA concentrations alone had little discriminant power in terms of MS(+) vs. MS(−) individuals. However, Phe was crucial in the multifactorial MS classification model with good discrimination power. Although adipocytokines and INS are commonly considered to be the classical MS contributors [48, 49], and ADI and LEP have already been suggested to be valuable MS biomarkers [50, 51], our study is the first to show Phe is useful for MS diagnostic model construction. Moreover, in this model Phe had a greater effect on the diagnostic index value than did ADI and LEP, and had an effect equal to that of INS. It should be mentioned that Phe concentration was already identified as valuable component of MS prediction model [1]; surprisingly in that study Phe level was found to be decreased in patients with MS but this inconsistency with previous studies [5, 15] was not explained.

The classification properties of the *MS screen test* exceeded many of the models published to date, which were based on individual adipocytokines concentrations [50–53] or HOMA IR [54, 55]. Our results support the hypothesis that BCAA and AAA profile plays an important role in disturbances in MS.

The *MS screen* test accounts for multifactorial pathogenesis of MS and, as far as we know, is the first comprehensive model that integrates representatives of its main pathological processes. Good classification properties, ability to stratify the disease risk, and potential to be used for monitoring of disease progression or positive treatment outcomes are needs that must be met by the next early MS biomarker. *MS screen test* results can be achieved with just one blood sample; thus, the test it could be a powerful tool in preventive medicine.

However, it must be noted that the *MS screen test* was built using data from a relative small group of participants and, as such, requires validation using data from additional populations. In addition, an assessment of the prediction value for DM2 and CVD should be undertaken.

Since clear differences between male and female BCAA catabolism have been previously established [56, 57], only males were recruited to the study. However this choice limits the clinical utility of *MS screen test*. In addition, we acknowledge other limitations of our study: firstly, when analyzing AA and obesity, indirect imprecise measures of VF assessment were used [39, 58]; secondly, the possible influence of physical activity on the BCAA catabolic pathway [59] was not considered. The metabolism of BCAA is very effective, and exceeds daily consumption [60]. Previous studies showed independence of fasting plasma BCAAs on diet [61]. However, regarding published work demonstrating BCAA metabolism impairment and influence of other macronutrients [9], it may also have been beneficial to consider the effect of diet on AA profile.

In summary, we demonstrated the existence of specific MS disturbances in BCAA and AAA profile, which may be a part of CMD pathogenic processes and involve novel biomarkers. Although their basis and role is yet to be fully elucidated, the altered profile appears not to be a direct result of increased body/fat and muscle mass. The results presented herein did not confirm the existence of close association with insulin sensitivity impairment with respect to glucose metabolism. Finally, the novel MS diagnostic model developed and presented herein should be validated in future studies.

## References

[1] Pujos-Guillot E, Brandolini M, Pétéra M, Grissa D, Joly C, Lyan B, Herquelot Eacute, Czernichow S, Zins M, Goldberg M, Comte B (2017). Systems metabolomics for prediction of metabolic syndrome. J. Proteome Res..

[2] O’Neill S, Bohl M, Gregersen S, Hermansen K, O’Driscoll L (2016). Blood-based biomarkers for metabolic syndrome. Trends Endocrinol. Metab..

[3] O.A.H. Jones (ed), *Metabolomics and Systems Biology in Human Health and Medicine. CABI (2014) Roberts, L.D.: Type 2 DIabetes Mellitus and the Metabolic Syndrome*, Metabolomics and Systems Biology in Human Health and Medicine (CABI: USA, 2014), pp. 141–153

[4] Batch BC, Hyland K, Svetkey LP (2014). Branch chain amino acids: biomarkers of health and disease. Curr. Opin. Clin. Nutr. Metab. Care.

[5] Batch BC, Shah SH, Newgard CB, Turer CB, Haynes C, Bain JR, Muehlbauer M, Patel MJ, Stevens RD, Appel LJ, Newby LK, Svetkey LP (2013). Branched chain amino acids are novel biomarkers for discrimination of metabolic wellness. Metabolism.

[6] Weng L, Quinlivan E, Gong Y, Beitelshees AL, Shahin MH, Turner ST, Chapman AB, Gums JG, Johnson JA, Frye RF, Garrett TJ, Cooper-DeHoff RM (2015). Association of branched and aromatic amino acids levels with metabolic syndrome and impaired fasting glucose in hypertensive patients. Metab. Syndr. Relat. Disord..

[7] Nagao K, Yamakado M (2016). The role of amino acid profiles in diabetes risk assessment. [Review]. Curr. Opin. Clin. Nutr. Metab. Care.

[8] Newgard CB, An J, Bain JR, Muehlbauer MJ, Stevens RD, Lien LF, Haqq AM, Shah SH, Arlotto M, Slentz CA, Rochon J, Gallup D, Ilkayeva O, Wenner BR, Yancy WS, Eisenson H, Musante G, Surwit RS, Millington DS, Butler MD, Svetkey LP (2009). A branched-chain amino acid-related metabolic signature that differentiates obese and lean humans and contributes to insulin resistance. Cell Metab..

[9] Newgard CB (2012). Interplay between lipids and branched-chain amino acids in development of insulin resistance. Cell Metab..

[10] Adeva MM, Calviño J, Souto G, Donapetry C (2011). Insulin resistance and the metabolism of branched-chain amino acids in humans. Amino Acids.

[11] Magnusson M, Lewis GD, Ericson U, Orho-Melander M, Hedblad B, Engström G, Ostling G, Clish C, Wang TJ, Gerszten RE, Melander O (2013). A diabetes-predictive amino acid score and future cardiovascular disease. Eur. Heart J..

[12] Wang TJ, Larson MG, Vasan RS, Cheng S, Rhee EP, McCabe E, Lewis GD, Fox CS, Jacques PF, Fernandez C, O’Donnell CJ, Carr SA, Mootha VK, Florez JC, Souza A, Melander O, Clish CB, Gerszten RE (2011). Metabolite profiles and the risk of developing diabetes. Nat. Med..

[13] Yamakado M, Nagao K, Imaizumi A, Tani M, Toda A, Tanaka T, Jinzu H, Miyano H, Yamamoto H, Daimon T, Horimoto K, Ishizaka Y (2015). Plasma free amino acid profiles predict four-year risk of developing diabetes, metabolic syndrome, dyslipidemia, and hypertension in Japanese population. Sci. Rep..

[14] McCormack SE, Shaham O, McCarthy MA, Deik AA, Wang TJ, Gerszten RE, Clish CB, Mootha VK, Grinspoon SK, Fleischman A (2013). Circulating branched-chain amino acid concentrations are associated with obesity and future insulin resistance in children and adolescents. Pediatr. Obes..

[15] Wiklund PK, Pekkala S, Autio R, Munukka E, Xu L, Saltevo J, Cheng S, Kujala UM, Alen M, Cheng S (2014). Serum metabolic profiles in overweight and obese women with and without metabolic syndrome. Diabetol. Metab. Syndr..

[16] Wu G (2009). Amino acids: metabolism, functions, and nutrition. Amino Acids.

[17] Hundal HS, Taylor PM (2009). Amino acid transceptors: gate keepers of nutrient exchange and regulators of nutrient signaling. Am. J. Physiol. - Endocrinol. Metab..

[18] Yoshizawa F (2012). New therapeutic strategy for amino acid medicine: notable functions of branched chain amino acids as biological regulators. J. Pharmacol. Sci..

[19] Lu J, Xie G, Jia W, Jia W (2013). Insulin resistance and the metabolism of branched-chain amino acids. Front. Med..

[20] C.X. Wang, F.F. Guo, Branched chain amino acids and metabolic regulation. Chin. Sci. Bull. **58**, 1228–1235 (2013)

[21] Fernstrom JD (2013). Large neutral amino acids: dietary effects on brain neurochemistry and function. Amino Acids.

[22] Choi S, DiSilvio B, Fernstrom MH, Fernstrom JD (2009). Meal ingestion, amino acids and brain neurotransmitters: Effects of dietary protein source on serotonin and catecholamine synthesis rates. Physiol. Behav..

[23] Coppola A, Wenner BR, Ilkayeva O, Stevens RD, Maggioni M, Slotkin TA, Levin ED, Newgard CB (2013). Branched-chain amino acids alter neurobehavioral function in rats. Am. J. Physiol. Endocrinol. Metab..

[24] Alberti KGMM, Eckel RH, Grundy SM, Zimmet PZ, Cleeman JI, Donato KA, Fruchart JC, James WPT, Loria CM, Smith SC (2009). Harmonizing the metabolic syndrome a joint interim statement of the international diabetes federation task force on epidemiology and prevention; national heart, lung, and blood institute; American heart association; world heart federation; international atherosclerosis society; and international association for the study of obesity. Circulation.

[25] Szurkowska M, Szafraniec K, Gilis-Januszewska A, Szybiński Z, Huszno B (2005). [Insulin resistance indices in population-based study and their predictive value in defining metabolic syndrome]. Przegląd Epidemiol..

[26] EZfaast, https://www.phenomenex.com/Products/AminoAcidDetail/EZfaast. Accessed 29 Aug 2017

[27] Seibert R, Abbasi F, Hantash FM, Caulfield MP, Reaven G, Kim SH (2015). Relationship between insulin resistance and amino acids in women and men. Physiol. Rep..

[28] Cynober LA (2002). Plasma amino acid levels with a note on membrane transport: characteristics, regulation, and metabolic significance. Nutrition.

[29] Adams SH (2011). Emerging perspectives on essential amino acid metabolism in obesity and the insulin-resistant state. Adv. Nutr. Adv. Nutr. Bethesda Md..

[30] Brown MS, Goldstein JL (2008). Selective versus total insulin resistance: a pathogenic paradox. Cell Metab..

[31] Cook JR, Langlet F, Kido Y, Accili D (2015). Pathogenesis of selective insulin resistance in isolated hepatocytes. J. Biol. Chem..

[32] Tan SX, Fisher-Wellman KH, Fazakerley DJ, Ng Y, Pant H, Li J, Meoli CC, Coster ACF, Stöckli J, James DE (2015). Selective insulin resistance in adipocytes. J. Biol. Chem..

[33] Würtz P, Soininen P, Kangas AJ, Rönnemaa T, Lehtimäki T, Kähönen M, Viikari JS, Raitakari OT, Ala-Korpela M (2013). Branched-chain and aromatic amino acids are predictors of insulin resistance in young adults. Diabetes Care.

[34] Giesbertz P, Daniel H (2016). Branched-chain amino acids as biomarkers in diabetes. Curr. Opin. Clin. Nutr. Metab. Care.

[35] Tom A, Nair KS (2006). Assessment of branched-chain amino acid status and potential for biomarkers. J. Nutr..

[36] She P, Van Horn C, Reid T, Hutson SM, Cooney RN, Lynch CJ (2007). Obesity-related elevations in plasma leucine are associated with alterations in enzymes involved in branched-chain amino acid metabolism. Am. J. Physiol. Endocrinol. Metab..

[37] B. Laferrère, K. Pietiläinen, Y. Boirie, in *Weight Loss and Branched Chain Amino Acids and Their Metabolites,* ed. by R. Rajendram, V.R. Preedy, V.B. Patel. Branched Chain Amino Acids in Clinical Nutrition: Volume 2 (Humana Press, Springer Science+Business Media, New York: USA, 2015), pp. 251–262

[38] Lackey DE, Lynch CJ, Olson KC, Mostaedi R, Ali M, Smith WH, Karpe F, Humphreys S, Bedinger DH, Dunn TN, Thomas AP, Oort PJ, Kieffer DA, Amin R, Bettaieb A, Haj FG, Permana P, Anthony TG, Adams SH (2013). Regulation of adipose branched-chain amino acid catabolism enzyme expression and cross-adipose amino acid flux in human obesity. Am. J. Physiol. Endocrinol. Metab..

[39] Yamakado M, Tanaka T, Nagao K, Ishizaka Y, Mitushima T, Tani M, Toda A, Toda E, Okada M, Miyano H, Yamamoto H (2012). Plasma amino acid profile is associated with visceral fat accumulation in obese Japanese subjects. Clin. Obes..

[40] M. Cansev, R.J. Wurtman, in *4 Aromatic Amino Acids in the Brain*, ed. by A. Lajtha, S.S. Oja, A. Schousboe, P. Saransaari. Handbook of Neurochemistry & Molecular Neurobiology: Amino Acids and Peptides in the Nervous System (Springer US: USA, 2007), pp. 59–97

[41] Skurk T, Rubio-Aliaga I, Stamfort A, Hauner H, Daniel H (2010). New metabolic interdependencies revealed by plasma metabolite profiling after two dietary challenges. Metabolomics.

[42] Koren MS, Purnell JQ, Breen PA, Matthys CC, Callahan HS, Meeuws KE, Burden VR, Weigle DS (2007). Changes in plasma amino acid levels do not predict satiety and weight loss on diets with modified macronutrient composition. Ann. Nutr. Metab..

[43] Fernstrom JD, Langham KA, Marcelino LM, Irvine ZLE, Fernstrom MH, Kaye WH (2013). The ingestion of different dietary proteins by humans induces large changes in the plasma tryptophan ratio, a predictor of brain tryptophan uptake and serotonin synthesis. Clin. Nutr..

[44] Wurtman RJ, Wurtman JJ (1995). Brain serotonin, carbohydrate-craving, obesity and depression. Obes. Res.

[45] Blouet C, Jo YH, Li X, Schwartz GJ (2009). Mediobasal hypothalamic leucine sensing regulates food intake through activation of a hypothalamus–brainstem circuit. J. Neurosci..

[46] Carreiro AL, Dhillon J, Gordon S, Jacobs AG, Higgins KA, McArthur BM, Redan BW, Rivera RL, Schmidt LR, Mattes RD (2016). The macronutrients, appetite and energy intake. Annu. Rev. Nutr..

[47] Melnik BC (2012). Leucine signaling in the pathogenesis of type 2 diabetes and obesity. World J. Diabetes.

[48] Maury E, Brichard SM (2010). Adipokine dysregulation, adipose tissue inflammation and metabolic syndrome. Mol. Cell. Endocrinol..

[49] Eckel RH, Grundy SM, Zimmet PZ (2005). The metabolic syndrome. The Lancet.

[50] Mirza S, Qu HQ, Li Quan, Martinez, Rentfro PJ, McCormick AR, Fisher-Hoch JB, Adiponectin/leptin SP (2011). ratio and metabolic syndrome in a Mexican American population. Clin. Invest. Med..

[51] Zhuo Q, Wang Z, Fu P, Piao J, Tian Y, Xu J, Yang X (2009). Comparison of adiponectin, leptin and leptin to adiponectin ratio as diagnostic marker for metabolic syndrome in older adults of Chinese major cities. Diabetes Res. Clin. Pract..

[52] Fujikawa R, Ito C, Tsuboi A (2015). Is the screening of metabolic syndrome using adiponectin possible?. Diabetol. Int..

[53] Hara K, Horikoshi M, Yamauchi T, Yago H, Miyazaki O, Ebinuma H, Imai Y, Nagai R, Kadowaki T (2006). Measurement of the high-molecular weight form of adiponectin in plasma is useful for the prediction of insulin resistance and metabolic syndrome. Diabetes Care.

[54] Gayoso-Diz P, Otero-González A, Rodriguez-Alvarez MX, Gude F, García F, Francisco AD, Quintela AG (2013). Insulin resistance (HOMA-IR) cut-off values and the metabolic syndrome in a general adult population: effect of gender and age: EPIRCE cross-sectional study. BMC Endocr. Disord..

[55] Esteghamati A, Ashraf H, Khalilzadeh O, Zandieh A, Nakhjavani M, Rashidi A, Haghazali M, Asgari F (2010). Optimal cut-off of homeostasis model assessment of insulin resistance (HOMA-IR) for the diagnosis of metabolic syndrome: third national surveillance of risk factors of non-communicable diseases in Iran (SuRFNCD-2007). Nutr. Metab..

[56] Patel MJ, Batch BC, Svetkey LP, Bain JR, Turer CB, Haynes C, Muehlbauer MJ, Stevens RD, Newgard CB, Shah SH (2013). Race and sex differences in small-molecule metabolites and metabolic hormones in overweight and obese adults. OMICS J. Integr. Biol..

[57] Newbern D, Gumus Balikcioglu P, Balikcioglu M, Bain J, Muehlbauer M, Stevens R, Ilkayeva O, Dolinsky D, Armstrong S, Irizarry K, Freemark M (2014). Sex differences in biomarkers associated with insulin resistance in obese adolescents: metabolomic profiling and principal components analysis. J. Clin. Endocrinol. Metab..

[58] Tchernof A, Després JP (2013). Pathophysiology of human visceral obesity: an update. Physiol. Rev..

[59] Kujala UM, Mäkinen VP, Heinonen I, Soininen P, Kangas AJ, Leskinen TH, Rahkila P, Würtz P, Kovanen V, Cheng S, Sipilä S, Hirvensalo M, Telama R, Tammelin T, Savolainen MJ, Pouta A, O’Reilly PF, Mäntyselkä P, Viikari J, Kähönen M, Lehtimäki T, Elliott P, Vanhala MJ, Raitakari OT, Järvelin MR, Kaprio J, Kainulainen H, Ala-Korpela M (2013). Long-term leisure-time physical activity and serum metabolome. Circulation.

[60] Elango R, Ball RO, Pencharz PB (2015). Tolerability of Leucine in Humans. In: Branched Chain Amino Acids in Clinical Nutrition.

[61] Noguchi Y, Zhang QW, Sugimoto T, Furuhata Y, Sakai R, Mori M, Takahashi M, Kimura T (2006). Network analysis of plasma and tissue amino acids and the generation of an amino index for potential diagnostic use. Am. J. Clin. Nutr..

